# MicroRNA-25-3p promotes cisplatin resistance in Non-small-cell lung carcinoma (NSCLC) through adjusting PTEN/PI3K/AKT route

**DOI:** 10.1080/21655979.2021.1939577

**Published:** 2021-07-16

**Authors:** Butong Sun, Nanjun Hu, Dan Cong, Kang Chen, Jun Li

**Affiliations:** Department of Hematology and Oncology, China-Japan Union Hospital of Jilin University, Changchun City, China

**Keywords:** miR-25-3p, PTEN/PI3K/AKT, cisplatin resistance, NSCLC

## Abstract

MicroRNAs exert crucial effects in the drug resistance. The purpose of this research was to investigate the miR-25-3p effects on DDP resistance in NSCLC. We used RT-qPCR to evaluate the expression of miR-25-3p. Cell growth was determined using MTS assay. Cellular bio-activity was analyzed via Colony formation, Annexin V/PI, and Transwell assay. Luciferase reporter assay was used to determine miR-25-3p and PTEN binding. Western blot was used to determine PTEN, PI3K, *p*-AKT/AKT expression. In-vivo study was used to determine the effects of miR-25-3p on the tumor growth. Expression of miR-25-3p is increased in NSCLC cisplatin resistant A549 and H1299 cells. Furthermore, miR-25-3p mimic enhanced drug resistance, and accelerated cell invasion and metastasis. Moreover, miR-25-3p mimic resulted in the activation of PTEN/PI3K/AKT pathway. However, miR-25-3p inhibitors exhibited the opposite trend. We further identified PTEN as a potential target of miR-25-3p. PTEN knockout promoted cisplatin resistance, while PTEN mimic displayed opposite effects. Interestingly, miR-25-3p further boosted cisplatin resistance cells in vivo, and miR-25-3p inhibitors reduced the in-vivo tumor volume. MiR-25-3p/PTEN/PI3K/AKT axis might accelerate DDP tolerance in NSCLC, which may serve as a potential target for chemotherapy resistance in NSCLC.

## Graphical abstract

**Figure:**
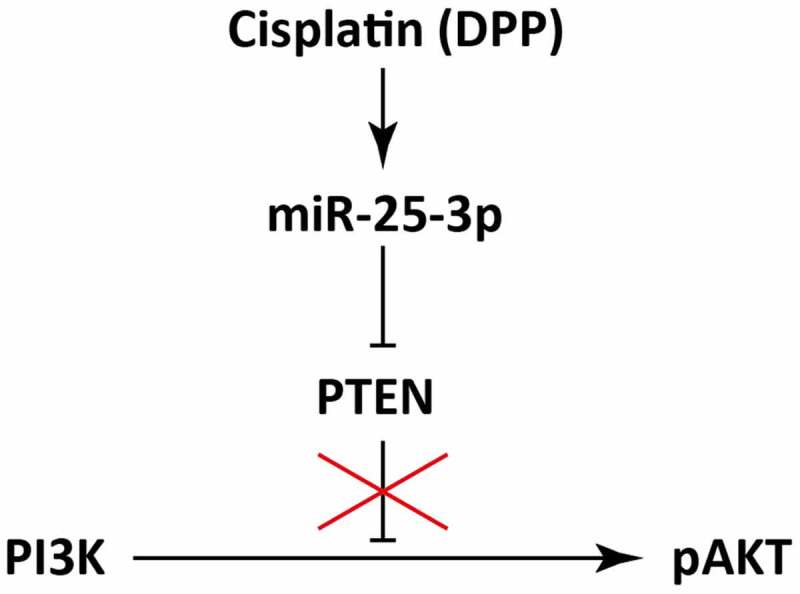

## 1. Introduction

Non-small cell lung cancer (NSCLC) is regarded as most acute and prevalent cancer [1]. So far, treatment for NSCLC remains chemotherapy-based [2]. Chemotherapy, as the preferred treatment means, exerts an important function in ameliorating the quality of life and prolonging the overall survival among NSCLC patients [3,4].

Cisplatin, also termed as DDP and considered as vulgaris antitumor drug, accelerates apoptosis via DNA damage and changes of cell metabolism [4]. However, it has been reported that cisplatin triggers several acute adverse reactions, including alopecia, vomiting, and myelosuppression [5]. Chemotherapy resistance mainly comprises primary drug resistance and acquired drug resistance [6]. Acquired drug resistance mianly displays tumor cells sensitivity to certain chemotherapeutic drugs decreased after several applications [7,8].

Many recent investigations suggested that PI3K/AKT signaling pathway play critical role in several diseases, including metabolic diseases and tumors by playing critical role in the cell proliferation [9,10]. Therefore, PI3K/AKT pathway inhibits the tumor development, and brings about poor prognosis of many cancers [11]. Several microRNAs (miRNAs), small non-coding RNAs (ncRNAs), have been identified as potential biomarkers for NSCLC [12]. Moreover, these miRNAs regulate the biological and cellular functions in NSCLC, thus playing a role in the pathogenesis of NSCLC [13]. Numerous miRNAs have been identified to be associated with PTEN/PI3K/AKT signaling pathway which play vital role in the survival of cancer patients [14,15]. Interestingly, miRNAs mediate the cisplatin resistance in NSCLC [16,17]. It has been studied that miR-26a level regulates AKT expression to counter DDP resistance [18]. Whereas, miRNA-17/1244 regulates cisplatin resistance in NSCLC via targeting TP53 and βR2 [19,20]. It has been previously investigated that miR-25-3p is upregulated in the cigarette smoke condensate (CSC) [21]. Moreover, miR-25-3p promotes invasion of human non-small cell lung cancer via CDH1 [22]. However, the exact role of miR-25-3p in cisplatin drug resistance mechanism in NSCLC remained unclear.

Based on this, our research attempted to inquire miR-25-3p expression in cisplatin resistance, so as to provide theoretical basis for improving the clinical efficacy of cisplatin chemotherapy in NSCLC.

## 2. Materials and methods

### 2.1. Cell culture and cisplatin treatment

A549 and H1299 cells were derived from the American culture library (Tongpai Biotechnology Co., Ltd). The cells were resuspended, and cultivated in RPMI1640 with 10% FBS at 37°C supplemented with 5% CO_2_. To obtain cisplatin resistant NSCLC cells, different concentrations of cisplatin (2, 4, 6, 8, 16, 32, 64) μmol/L were used for 6 h. Later, cells were used for subsequent assays.

### 2.2. qRT-PCR analysis

Trizol was used to isolate RNA. Then, monolithic RNA (Shanghai shanran Biotechnology Co., Ltd) kit was used to prepare the cDNA. SYBR Green Master Mix II (Adlai Biotechnology Co., Ltd) was used for RT-qPCR. U6 and GAPDH were used as internal control. 2-ΔΔCT approaches were utilized to analyze data.

### 2.3. MTS assay

Applied miR-25-3p mimics to transfect A549/DDP cells. Similarly, A549 cells were also treated with miR-25-3p inhibitor or NC inhibitor using liposome 2000 (Beijing wobison Technology Co., Ltd). After 72 h, the cell growth was assessed by MTS (Beijing Jinglei Technology Development Co., Ltd). Absorbance was measured via ELISA reader (Shandong Boke Biological Industry Co., Ltd).

### 2.4. Luciferase constructs assay

For luciferase assay, we inserted amplified DNA sequence into the psi-check2 vector. Briefly, we prepared the wildtype (WT) and Mutant (MUT) PTEN 3ʹ-UTR luciferase vectors. Then, cells were transfected with these vectors in presence of miR-25-3p mimics or NC, and the luciferase activity was determined.

### 2.5. Colony formation assay

A total of 400 cells/well were cultured into 6-well plates for 14 d, and treated with miR-25-3p mimics or inhibitors. Then, cells were stained using crystal violet solution (Qingdao jieshikang Biotechnology Co., Ltd). To calculate the data, a cluster of 50 cells was regarded as a colony.

### 2.6. Transwell assay

First, 100 μL Matt Riegel gel was added into serum-free culture medium. Then, 100 μL of cellular suspension and 200 μL serum-free culture media was mixed in Transwell upper chamber. Transwell lower chamber was filled with 300 μL complete culture medium containing 0.05% fetal bovine serum, and incubated together for 2 d. Later, upper chamber was fixed via paraformaldehyde and dyed via crystal violet. Polyester films were gained from the upper room base. The field was acquired under high microscope (× 400).

### 2.7. Flow cytometry test

The cells were transfected with miR-25-3p mimics or inhibitors. V-FITC and PI were added into cell suspension, and incubated at 4°C overnight. The flow cytometry was applied to detect apoptosis rate.

### 2.8. Western blot assays

Total protein was extracted using RIPA buffer, and quantified using BCA kit. Then, protein samples (20 μg) were run using SDS-PAGE. Subsequently, the proteins were transferred to PVDF membrane, blocked with 5% skim milk, and incubated with specific first antibody at 4°C for overnight. Finally, the membranes were washed, and further incubated with the horseradish peroxidase coupled secondary antibody. Finally, the protein bands were identified by using the enhanced chemiluminescence kit (Nanjing novozan Biotechnology Co., Ltd). Β-actin was used as internal control.

### 2.9. Tumor formation in nude mice

Mice were acquired from animal laboratory. All experiments related to the use of animals were permitted through the ethics committee. 0.5 × 107 cells were inoculated under the soft skin of the right forelimb on the back of nude mice. Cisplatin (3 mg/kg) was injected i.p. to mice once every two weeks. Later, the animals were sacrificed, and the tumor volume and weight were calculated. The animal experiment research protocol was approved by the Ethics Committee of China-Japan Union Hospital of Jilin University and performed in accordance with the ‘Guidelines for the care and use of experimental animals.’

### 2.10. Statistical analysis

Data were exhibited as X ± S and were analyzed by *t*-test between different groups. *P* < 0.05 was considered as statistically significant difference.

## 3. Results

### 3.1. Expression of miR-25-3p is increased during cisplatin resistance in NSCLC

**Figure 1.**
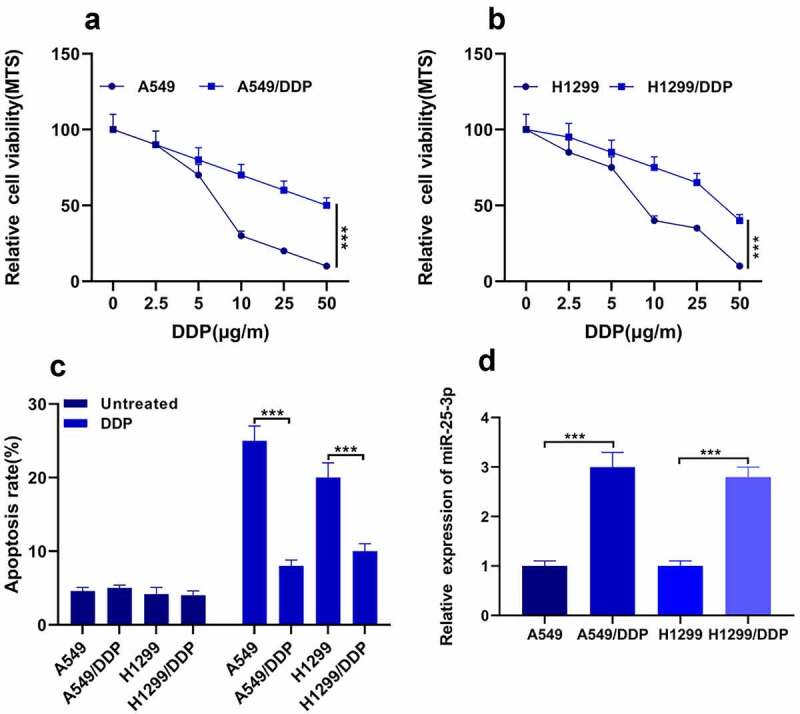
miR-25-3p was highly expressed in NSCLC. (a–b) MTS was utilized for determining two cells’ sensitivity to DDP. (c) Flow cytometry was utilized for measuring apoptosis. (d) RT-PCR found that miR-25-3p expression in two cells was memorably lower. *** *P* < 0.001

It was previously studied that miR-25-3p is upregulated in the cigarette smoke condensate (CSC) [21], and promotes invasion of human non-small cell lung cancer via CDH1 [22]. However, the exact role of miR-25-3p in cisplatin drug resistance mechanism in NSCLC remained unclear. In this study, firstly we detected A549 DDP cells sensitivity to DDP via MTS methods; see Figure 1a for more details. Our outcomes found that A549/H1299 DDP drug-fast was less. Surprisingly, we also found that H1299 cells displayed weaker strength to DDP (Figure 1b). Moreover, the apoptosis rate was decreased after the DDP treatment (Figure 1c). Finally, we used RT-qPCR to analyze the miR-25-3p expression. Our results demonstrated that expression of miR-25-3p was significantly increased in both cell lines after DDP treatment (Figure 1d). This concluded that the expression of miR-25-3p is increased during cisplatin resistance in NSCLC.

### 3.2. miR-25-3p mimic promotes cisplatin resistance and migration

**Figure 2.**
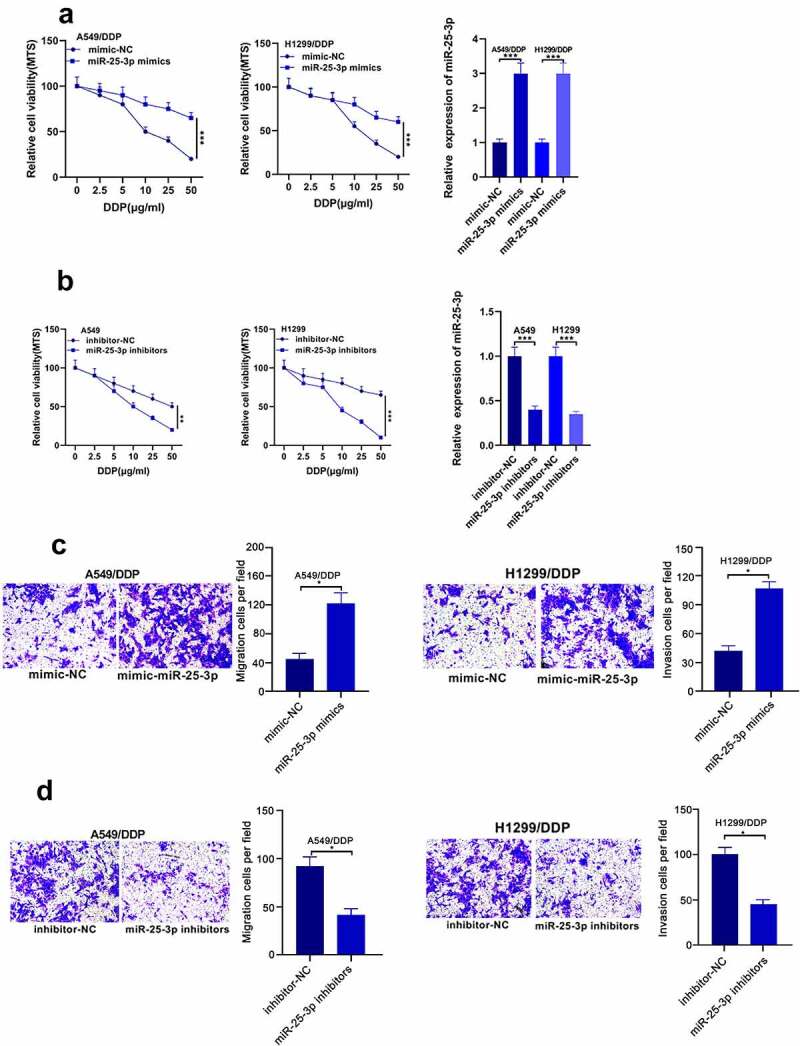
miR-25-3p lowered DDP drug-fast as well as A549/DDP progress. (a–b) MTS was applied to detect miR-25-3p level via miR-25-3p mimic/inhibitor treatment. (c-d) The A549/DDP cells’ movement was determined via miR-25-3p mimic/inhibitor transfection. **/***P < 0.01/0.001

Then, we probed whether miR-25-3p mimic could regulate DDP drug-fast and migration in A549 and H1299 cell lines. Our results showed that miR-25-3p mimics increase the cell proliferation and migration. Whereas miR-25-3p inhibitor displayed opposite trend (Figure 2a–d). These results implied that miR-25-3p play a role in promoting cisplatin resistance and NSCLC migration.

### 3.3. miR-25-3p mimic restrained DDP led apoptosis

**Figure 3.**
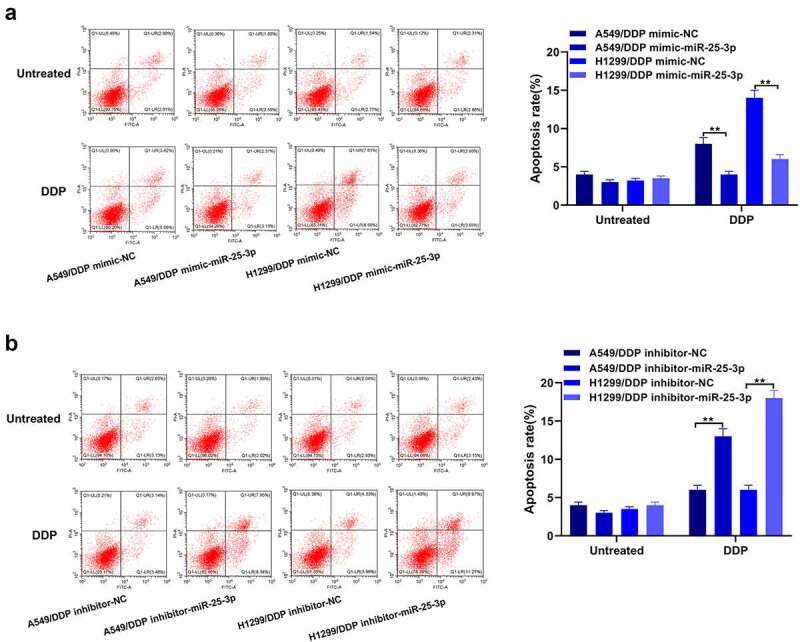
miR-25-3p over-expression controlled apoptosis. (a–b) Flow cytometry was adopted to detect apoptotic via miR-25-3p mimic/restrainer transfection. **P < 0.01

In order to validate the role of miR-25-3p in A549 and H1299 cells apoptosis, we performed the flow cytometry. From the Figure 3a, our team discovered that miR-25-3p over-expression exerted inhibitory effects on the cisplatin-induced apoptosis. On the contrary, miR-25-3p inhibitor showed opposite trends (Figure 3b). This shows that mir-25-3p restrained DDP-induced apoptosis.

### 3.4. PTEN deem as a target for miR-25-3p

**Figure 4.**
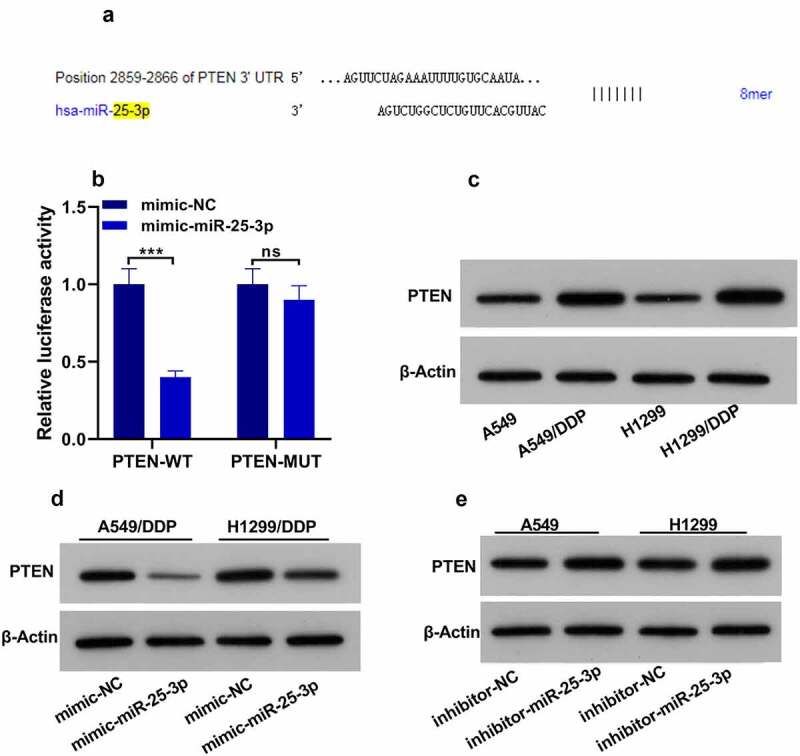
PTEN was called as miR-25-3p spot. (a) The bioinformatics database shows that miR-25-3p contains the conservative binding site of PTEN. (b) The cell activity was detected by fluorescein. (c) Western blot was utilized for measuring PTEN expression. (d–e) The miR-25-3p mimic/restrainer plasmid was infected into two cells for 24 h. Then, PTEN expression was assessed. ** *P* < 0.01

According to the target prediction tools, PTEN was found as a potential target of miR-25-3p (Figure 4a). Then we used the luciferase assay, and we found that luciferase activity was significantly decreased in the presence of PTEN WT plasmid and miR-25-3p mimics (Figure 4b). However, PTEN-MUT transfection did not show any change in the relative luciferase activity. Moreover, A549/H1299 DDP displayed higher expression of PTEN (Figure 4c). Interestingly, miR-25-3p mimic significantly decreased PTEN expression. Nevertheless, miR-25-3p inhibitors displayed the opposite effect (Figure 4d–e). These results showed that miR-25-3p targets the 3ʹ UTR of PTEN, and regulates PTEN expression.

### 3.5. miR-25-3p participates in NSCLC drug-fast via PTEN/PI3K/AKT signaling pathway

**Figure 5.**
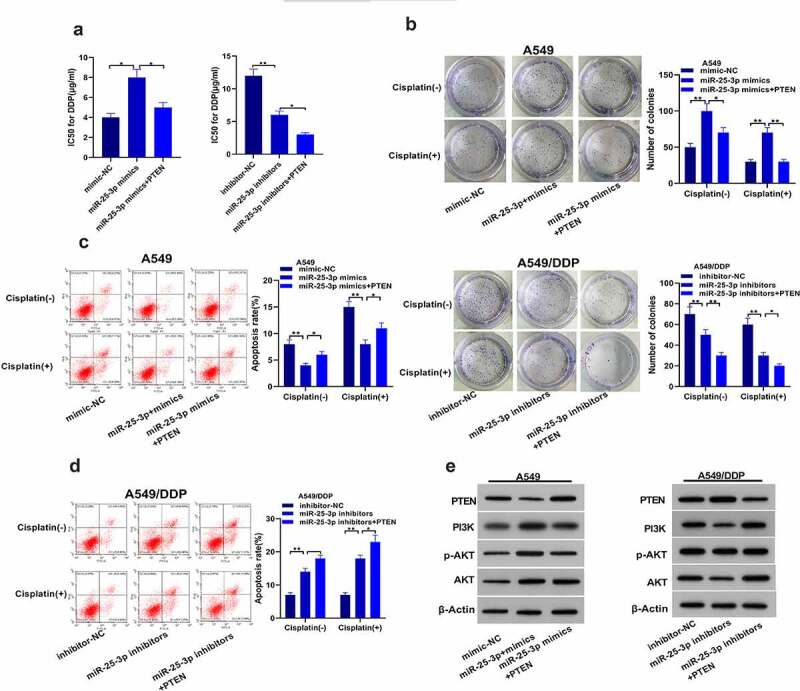
miR-25-3p enhanced chemoresistance of NSCLC cells. (a) miR-25-3p mimics ameliorated IC50 value. (b) Colony forming assay was used to detect miR-25-3p restrainer role on cell multiplication. (c) DDP induced apoptotic of A549 cells infected with miR-25-3p over-expression via flow cytometry. (d) Cisplatin induced apoptotic of A549/DDP cells infected with miR-25-3p inhibitor via flow cytometry. (e) After miR-25-3p mimic/inhibitor/PTEN cotransfected, our crew detected PTEN, PI3K, p-AKT and AKT levels. (-), not treated with cisplatin (+), treated with cisplatin

Our further results showed that miR-25-3p over-expression improved IC50. miR-25-3p high-expression/PTEN co transfection decreased this higher IC50. PTEN-overexpression further boosted miR-25-3p inhibitor effects on IC50 of A549/DDP cells (Figure 5a). Similarly, PTEN-overexpression partially deteriorated the effects of miR-25-3p on the colony forming ability in A549 cells. Surprisingly, miR-25-3p inhibitor/PTEN simultaneous-transfection displayed higher ability to inhibit colony formation (Figure 5b). Furthermore, PTEN overexpression substantially reversed the miR-25-3p resist-apoptotic effects. Besides, it further raised the apoptosis of A549/DDP cells which was induced by miR-25-3p inhibitor (Figure 5c–d). Finally, western blot analysis showed that miR-25-3p boosted PI3K expression as well as *p*-Akt/Akt proportion (Figure 5e).

### 3.6. miR-25-3p attended DDP drug-fast via animal test

**Figure 6.**
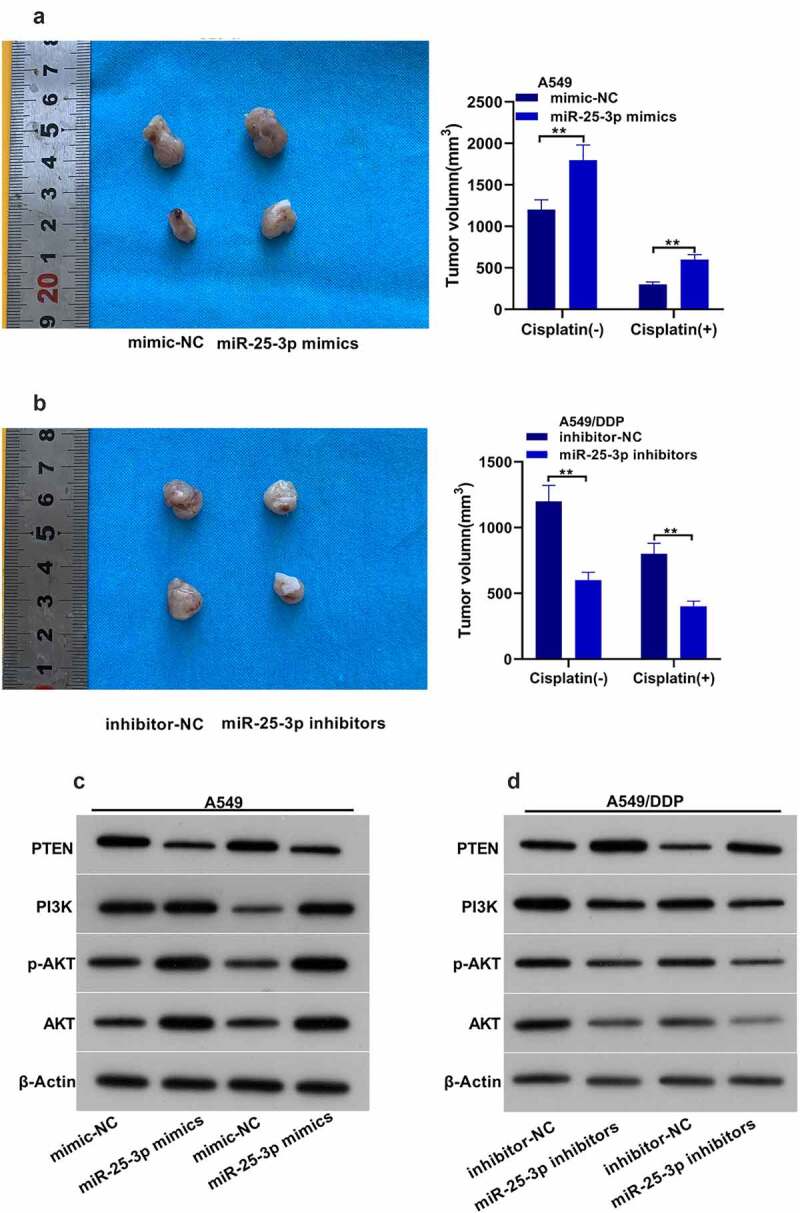
miR-25-3p partook DDP drug-fast via animal assay. (a–b) Mice tumor volume. (c–d) Western blot was used to detect PTEN, PI3K as well as p-AKT/AKT expression via miR-25-3p mimic/inhibitor transfection

Next, we found that the tumor volume in animals injected with miR-25-3p/A549 group was larger than that of miR-NC/A549 group (Figure 6a). The mean tumor volume in miR-25-3p inhibitors was evidently decreased (Figure 6b). Moreover, miR-25-3p mimics significantly decreased cisplatin-induced PTEN-expression. Besides, it also increased PI3K/*p*-AKT expression. However, miR-25-3p inhibitors displayed the opposite trend (Figure 6c–d).

## 4. Discussion

It is well known that with the aggravation of environmental pollution created by industrialization in China, the prevalence of NSCLC has been increased notably [23]. At the moment, cisplatin is regarded as the principal treatment for NSCLC [24]. Recent studies show that some patients may be susceptible to cisplatin during initial stages of treatment, while others may develop resistance gradually [4]. Unexpectedly, higher dose of cisplatin may also bring some serious side effects, and ultimately lower the life expectancy [25]. Therefore, we designed this study to expound DDP drug-fast for tumor to provide the individualized therapeutic strategy for NSCLC.

In this study, we explored that miR-25-3p expression is increased in NSCLC. Furthermore, over expression of miR-25-3p accelerated A549/DDP cell growth and metastasis, and restrained apoptosis.

Recent studies indicated that PTEN/PI3K pathway is intently related to tumor progression [26]. PI3K, which is composed of p85 and P110 subunits, acts as a phosphokinase. Whereas, Akt is known as the cardinal effector protein of PI3K [27]. The activation PI3K/AKT has been linked with the proliferation of cancerous cells and the expression of several tumor genes [28]. Our study validated that down regulation of PTEN reversed DDP drug-fast. Moreover, we also validated that miR-25-3p and PTEN exhibit a binding relationship. Therefore, we further found that miR-25-3p negatively regulates the expression of PTEN, and activates the PI3K/AKT signaling pathway, which might be considered as a latent mechanism for promoting cisplatin resistance in NSCLC.

It was found that PTEN inactivation raises NSCLC invasiveness and growth via PI3K/AKT/NFkB pathway [29]. Moreover, PTEN upregulation can minimize the NSCLC development and invasiveness [30]. Similar to our results, previous studies also identified miR-26 and miR-21 to be involved in the cell proliferation via adjusting PTEN [31,32]. However, in this study, we identified miR-25-3p/PTEN axis which affects NSCLC development in NSCLC cell lines and in-vivo tumor development.

## 5. Conclusion

In conclusion, our study reported that miR-25-3p expression is upregulated in cisplatin-resistant NSCLC cells. Moreover, overexpression of miR-25-3p enhances DDP drug-fast via regulating PTEN/PI3K/AKT signaling pathway. Therefore, inhibition of miR-25-3p may act as a novel strategy to overcome cisplatin resistance in NSCLC.

## Highlights

1. Expression of miR-25-3p is upregulated in cisplatin-resistant A549 and H1299 NSCLC cells.
2. MiR-25-3p regulates the NSCLC proliferation, and migration through PTEN/PI3K/AKT pathway.
3. Silencing miR-25-3p accelerates DDP tolerance, and decreases the tumor growth in vivo.
